# Anomalies of coronary artery origin at invasive angiography: a prospective study of prevalence, catheter utilisation, access strategy and procedural implications

**DOI:** 10.1186/s43044-026-00773-0

**Published:** 2026-08-10

**Authors:** Qayoom Yousuf, Aamir Rashid, Sameer Purra, Imran Hafeez, Bilal Syed, Aadil Amin Shah

**Affiliations:** Department of Cardiology, Sher-i-Kashmir Institute of Medical Sciences, Srinagar, India

**Keywords:** Anomalies of coronary artery origin, Coronary angiography, Percutaneous coronary intervention, Modified Angelini classification, Catheter selection

## Abstract

**Background:**

Anomalies of coronary artery origin are uncommon congenital variants that may pose a challenge to selective engagement at invasive coronary angiography (CAG). Although their prevalence and anatomical spectrum are well described, prospective data on the diagnostic and guide catheter utilisation to engage each anomaly subtype, procedural implications and on the access strategy adopted in current radial-first practice, remain limited.

**Methods:**

We prospectively enrolled 1,850 consecutive adults undergoing invasive CAG, with or without percutaneous coronary intervention (PCI). Anomalies of origin were defined by the modified Angelini classification; intrinsic anomalies and anomalies of termination were excluded. Baseline, angiographic, procedural data - including diagnostic and guide catheter use by subtype; and access strategy were compared between anomaly and non-anomaly groups.

**Results:**

An anomaly of origin was identified in 85 of 1,850 patients (4.59%; 95% CI 3.71–5.63%); under a restricted definition that excluded two anatomical variants not consistently classified as anomalies in earlier invasive-angiography series, prevalence was 1.30% (95% CI 0.83–1.92%). The commonest anomaly was ectopic (high or anterior) right coronary artery (RCA) origin from the right coronary cusp (*n* = 47; 55.3%), followed by separate ostia of left anterior descending (LAD) and left circumflex (LCx) arteries (*n* = 14; 16.5%) and anomalous LCx from the right coronary cusp or RCA (*n* = 14; 16.5%). PCI was performed in 52 of 85 anomaly patients (61.2%). Radial access was attempted in 67 patients: 53 (79.1%) completed radially, 14 (20.9%) required crossover to femoral; and 18 were attempted femorally. Anomaly patients required more diagnostic catheters per case (2.20 vs. 1.23), with the greatest burden in crossover cases (3.14 vs. 1.17 catheters); more access crossover (16.5% vs. 5.0%) and more guide catheter changes (44.2% vs. 6.5%; all *p* < 0.001),. No significant difference in major procedural complications was observed (4.7% vs. 2.7%; *p* = 0.43); however the study was not powered for rare procedural events.

## Introduction

Coronary artery anomalies are congenital variations in the origin, course, intrinsic anatomy or termination of the coronary arteries [1, 2]. The modified Angelini classification divides them into anomalies of origin and course, anomalies of intrinsic anatomy and anomalies of termination [3]. Anomalies of origin — an aberrant ostial location of an epicardial coronary artery — are the subset with the greatest practical relevance to the interventional cardiologist, since they directly influence catheter engagement, catheter selection and the risk of catheter-induced ostial trauma or dissection [4, 5]. Some anomalies, particularly those with an interarterial course, are also recognised non-atherosclerotic causes of sudden cardiac death in young adults [6–8]. Understanding of these anomalies has evolved substantially over the past decade, with contemporary reviews and society guidelines emphasising cross-sectional imaging, individualised risk stratification, and selective management of high-risk variants [9–11].

Reported prevalence of coronary anomalies varies widely, principally because of differences in case definition, study population and imaging modality [12–13]. Computed tomography (CT) coronary angiography series typically report higher figures than invasive-angiography series because CT readily identifies intrinsic anomalies — particularly myocardial bridging — that are under-diagnosed at invasive angiography [14–16]. Series including intrinsic anomalies are therefore not directly comparable with those confined to anomalies of origin. Several Indian centres have reported on coronary anomalies, with figures differing markedly across reports depending on definition [16–19]; however contemporary invasive-angiography data linking anomalies of origin to catheter utilisation, access strategy and procedural implications are limited.

Existing literature on catheter engagement in anomalous coronaries is predominantly case-based or narrative in character [9, 20, 21]. The present study extends the existing literature by providing prospective, subtype-resolved catheter and access data across the full spectrum of anomalies of coronary artery origin, in a large consecutive angiographic cohort from a contemporary radial-predominant practice.

## Methods

### Study design and population

This was a prospective, single-centre observational study of consecutive adult patients (≥ 18 years) undergoing diagnostic coronary angiography (CAG), with or without percutaneous coronary intervention (PCI), between October 2024 and October 2025. Institutional ethics committee approval and written informed consent were obtained. Given the observational nature of study all consecutive patients were enorolled with no formal power calculation.

### Coronary angiography and catheter strategy

Vascular access was radial by default; femoral access was preferred in ST-elevation myocardial infarction for primary PCI and in selected patients at the operator’s discretion. The access strategy was categorised as radial only, femoral only, or cross-over (from radial to femoral for either angiography or PCI). The default diagnostic catheter for radial access was a 5 F-Tiger (Terumo, Tokyo, Japan); for femoral access, 6 F Judkins Left (JL3.5) and Judkins Right (JR3.5) catheters were used. When standard catheters failed to engage a coronary ostium, additional shapes — Amplatz Left (AL0.75/1/2), Amplatz Right (AR1/2), Multipurpose (MP), Internal mammary artery (IMA), Contralateral left support (CLS), Extra back-up (EBU) and Three-dimensional right curve (3DRC) — were used until selective opacification was achieved [21]. Procedures were performed by a group of well-trained interventional cardiologists, each with more than five years of independent practice.

### Definition and classification of coronary anomalies

We restricted the definition of a coronary anomaly to anomalies of origin as defined by the modified Angelini classification [3] - that is, anomalies in which the ostium of an epicardial coronary artery arose from a non-standard ostial location. The following entities were included: (i) anomalous ectopic (high or anterior) origin of the right coronary artery (RCA) from the right coronary cusp (RCC); (ii) separate ostial origins of the left anterior descending (LAD) and left circumflex (LCx) arteries, representing congenital absence of the left main stem; (iii) anomalous origin of the LCx from the RCC or proximal RCA; (iv) anomalous origin of the RCA from the left coronary cusp (LCC); (v) anomalous origin of the RCA from the non-coronary cusp; and (vi) anomalous origin of the LAD from the RCA. For the ectopic (high or anterior) RCA subtype, a “high” origin was defined as an RCA ostium located at or above the sinotubular junction, and an “anterior” origin as an ostium located anterior to the mid-plane of the RCC; both required an aortic root injection or a non-standard catheter shape for selective engagement. Classification was based on invasive angiographic appearance in all cases.

Intrinsic anomalies (myocardial bridging, split RCA, dual LAD) and anomalies of termination (coronary–cameral fistulas) were excluded by design. This restriction was made because: (i) our primary objective was to evaluate the procedural and catheter-related implications of coronary anomalies, and it is anomalies of origin that directly affect ostial engagement and catheter selection; (ii) intrinsic anomalies are most reliably identified by CT coronary angiography or intravascular imaging and are under-diagnosed on routine invasive angiography [4, 14]; and (iii) restricting the analysis to anomalies of origin permits direct comparison with the classical international invasive-angiography literature [3, 5, 12, 22]. As our study was scoped to the procedural difficulty of engaging anomalous coronary ostia rather than to anatomical course characterisation or prognostication, CT coronary angiography was not performed routinely and was reserved for selected patients in whom characterisation of vessel course was clinically necessary. Anomalies were adjudicated by two experienced interventional cardiologists reading together in consensus, with disagreements resolved by discussion.

### Variables and statistical analysis

Variables recorded included age, sex, cardiovascular risk factors, clinical presentation (STEMI, NSTEMI, unstable angina, chronic coronary syndrome, or others), access strategy, diagnostic and guide catheters used, angiographic diagnosis and peri-procedural complications. Dyslipidaemia was defined as an LDL-cholesterol ≥ 130 mg/dL, total cholesterol ≥ 200 mg/dL, triglycerides ≥ 150 mg/dL, or ongoing lipid-lowering therapy. Hypertension was defined as a systolic blood pressure ≥ 140 mmHg or diastolic blood pressure ≥ 90 mmHg on two or more clinic measurements, or ongoing antihypertensive therapy. Diabetes mellitus was defined as a fasting plasma glucose ≥ 126 mg/dL, HbA1c ≥ 6.5%, or ongoing anti-diabetic medications. A “major procedural complication” was defined as any of the following occurring during the index procedure or within 24 h of it: (i) catheter-induced coronary dissection requiring intervention; (ii) periprocedural death (all-cause, irrespective of direct catheter attribution); (iii) any major bleeding meeting BARC type ≥ 3 [23]; and (iv) any event requiring urgent additional cardiac intervention. Complications were captured prospectively during the procedure and at hospital discharge. Continuous variables are presented as mean ± standard deviation and compared with Welch’s t-test; categorical variables as counts and percentages compared with χ⁴ (Fisher’s exact test where expected counts < 5). In addition to univariate comparisons, exploratory multivariable logistic regression was performed for access crossover with coronary anomaly, age, sex, clinical presentation and PCI performance as covariates. A two-tailed *p* < 0.05 was considered significant.

## Results

### Cohort characteristics and prevalence

A total of 1,850 consecutive patients were included; mean age 58.4 ± 11.2 years, 1,486 (80.3%) male. Hypertension (75.7%), smoking (52.4%), dyslipidaemia (36.5%) and diabetes (31.6%) were the commonest risk factors. An anomaly of coronary artery origin was identified in 85 patients (4.59%; 95% CI 3.71–5.63%). Under a restricted definition that excluded the two commonest benign anatomical variants — ectopic (high or anterior) origin of the RCA from the RCC (*n* = 47) and separate ostial origins of the LAD and LCx (*n* = 14) — the prevalence was 24/1,850 (1.30%; 95% CI 0.83–1.92%), in keeping with historical invasive-angiography series that used this narrower case definitions, for comparison [5, 12, 22].

### Baseline characteristics

Baseline characteristics are summarised in Table 1. There were no significant differences in age, sex or the prevalence of hypertension, diabetes or smoking; dyslipidaemia tended to be less common in the anomaly group (25.9% vs. 37.1%; *p* = 0.05). The indication for angiography in anomaly patients was STEMI in 52 (61.2%), NSTEMI in 12 (14.1%), unstable angina in 2 (2.4%), and chronic coronary syndrome or other in 19 (22.4%). Among non-anomaly patients, STEMI was the indication in 703 (39.8%), NSTEMI in 403 (22.8%), unstable angina in 47 (2.7%), and chronic coronary syndrome or other in 612 (34.7%); the anomaly group therefore had a higher proportion of STEMI presentations than the non-anomaly group (61.2% vs. 39.8%, *p* < 0.001).

**Table 1:** Baseline characteristics of patients with and without an anomaly of coronary artery origin

Variable	Anomaly (*n* = 85)	No anomaly (*n* = 1,765)	Overall (*N* = 1,850)	*P*
Age, years (mean ± SD)	59.0 ± 11.0	58.3 ± 11.3	58.4 ± 11.2	0.59
Male, n (%)	71 (83.5)	1,415 (80.2)	1,486 (80.3)	0.53
Hypertension, n (%)	69 (81.2)	1,332 (75.5)	1,401 (75.7)	0.28
Diabetes mellitus, n (%)	20 (23.5)	565 (32.0)	585 (31.6)	0.13
Dyslipidaemia, n (%)	22 (25.9)	654 (37.1)	676 (36.5)	0.05
Current smoking, n (%)	50 (58.8)	919 (52.1)	969 (52.4)	0.27
STEMI, n (%)	52 (61.2)	703 (39.8)	755 (40.8)	< 0.001
NSTEMI, n (%)	12 (14.1)	403 (22.8)	415 (22.4)	0.06
Unstable angina, n (%)	2 (2.4)	47 (2.7)	49 (2.6)	1.00
Chronic coronary syndrome / other, n (%)	19 (22.4)	612 (34.7)	631 (34.1)	0.02

*SD* standard deviation, *STEMI* ST-elevation myocardial infarction, *NSTEMI* non-ST-elevation myocardial infarction

### Spectrum of anomalies of origin

**Fig. 1:**
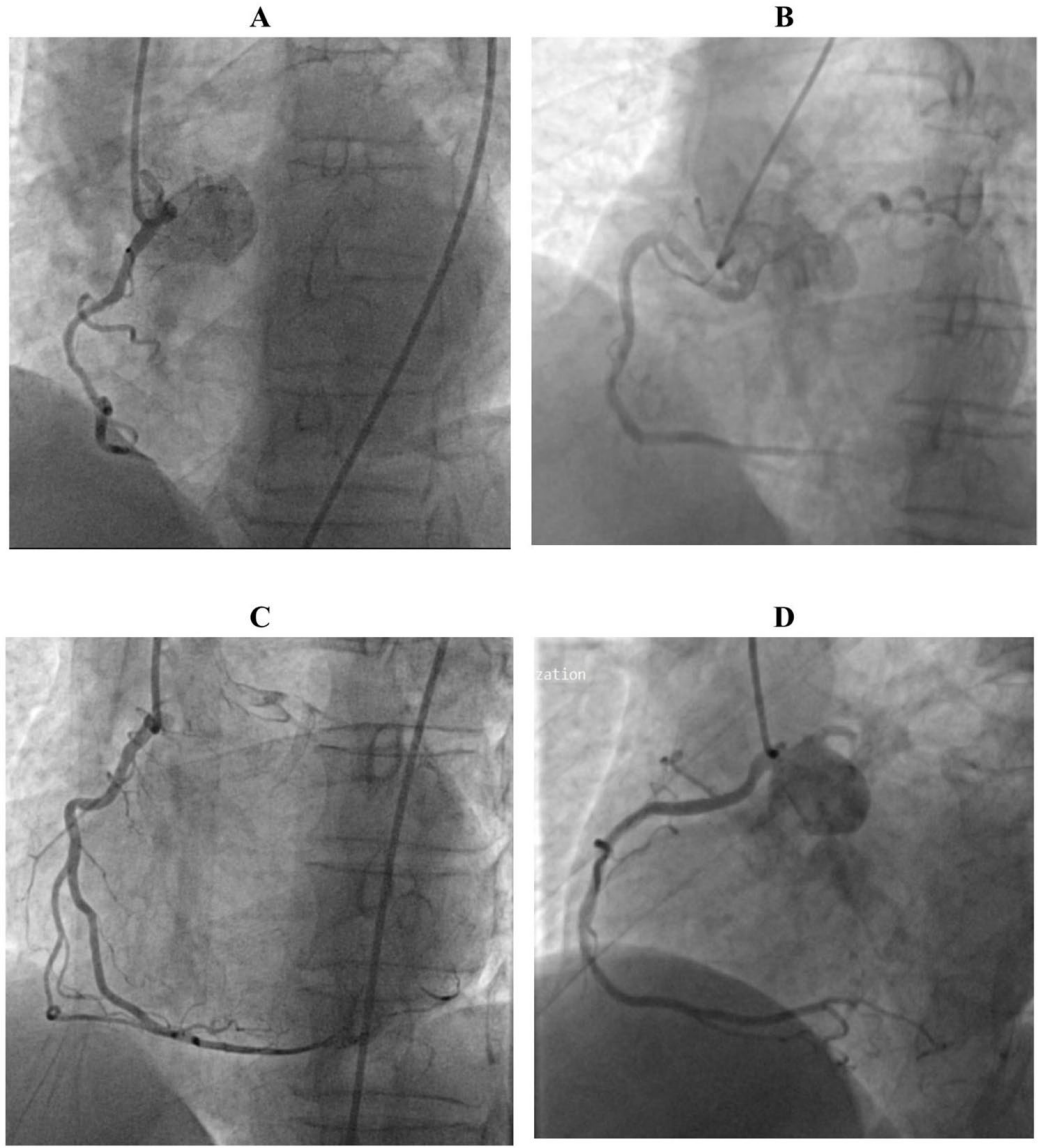
Anomalous ectopic (high or anterior) origin of the RCA from the right coronary cusp. (in LAO projection). **A–C** Anterior origin of the RCA. **D** Anterior and high origin of the RCA

**Fig. 2:**
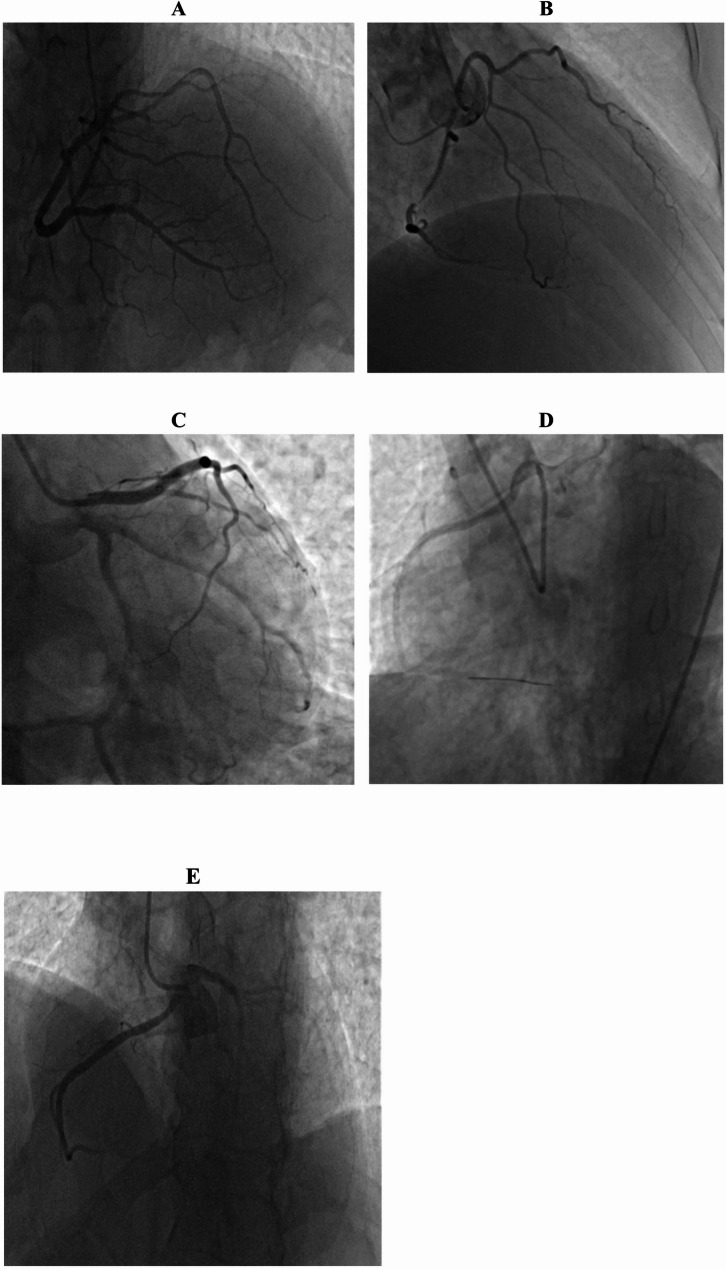
Anomalies of coronary artery origin. **A** LAD from the RCA (RAO 30*°*/Caudal 30*°)*. **B** LAD from the RCA (RAO 40°). **C** Separate ostial origins of the LAD and LCx (absent left main stem) in RAO 30*°*/Caudal 30*°*. **D** Common origin of the RCA and left main from a single ostium, in the left coronary cusp (LAO 30*°*). **E** Separate origins of the RCA and left main from the left coronary cusp in LAO 30*°*/Cranial 30*°*

**Fig. 3:**
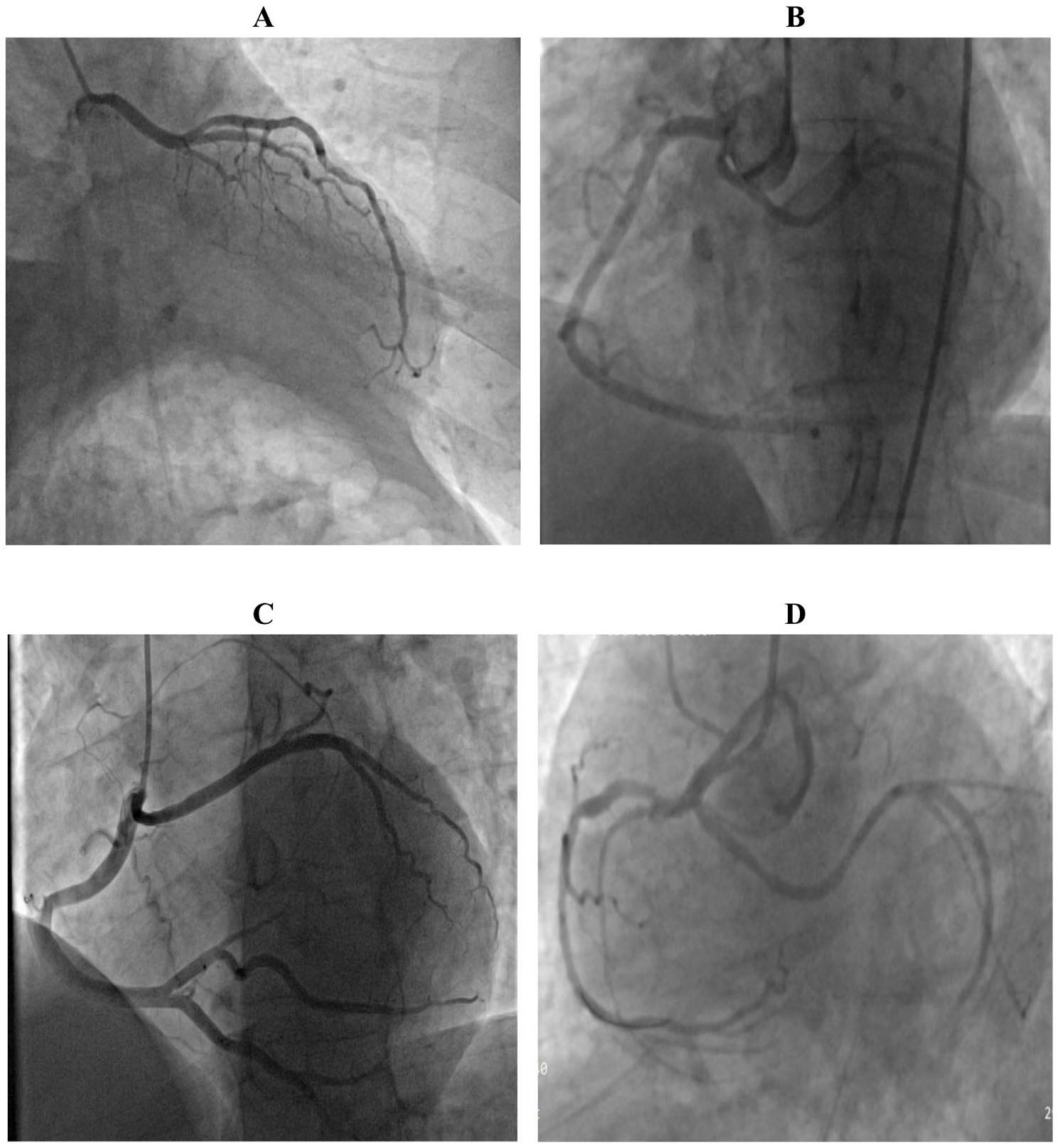
Anomalous origin of the left circumflex artery (LCx) from the RCC or RCA. **A** Left coronary injection (RAO 30° / caudal 25°) showing a long left main continuing as the LAD **B** Right injection (LAO 30°) demonstrating the RCA and a separate anomalous LCx arising from the same right cusp. **C** Anomalous LCx arising from the ostial RCA (RAO 30°). **D** Anomalous LCx arising from the proximal RCA (RAO 40°)

The spectrum of anomalies is shown in Table 2. The commonest anomaly was an ectopic (high or anterior) origin of the RCA from the RCC, identified in 47 patients (55.3% of anomalies; 2.54% of the overall cohort; Fig. 1). This was followed by separate ostial origins of the LAD and LCx in fourteen patients (16.5%; Fig. 2) and an anomalous origin of the LCx from the RCC or proximal RCA in fourteen patients (16.5%; Fig. 3). Less common variants included an anomalous origin of the RCA from the LCC (*n* = 6; 7.1%; Fig. 3), the RCA from the non-coronary cusp (*n* = 2; 2.4%) and an anomalous LAD arising from the RCA (*n* = 2; 2.4%; Fig. 3).

**Table 2:** Spectrum of anomalies of coronary artery origin

Anomaly of origin	*N*	% of anomalies	% of cohort (*N* = 1,850)
Anomalous ectopic (high or anterior) origin of RCA from right coronary cusp	47	55.3	2.54
Separate ostia of LAD and LCx (absent left main)	14	16.5	0.76
Anomalous LCx from right coronary cusp or RCA	14	16.5	0.76
RCA from left coronary cusp	6	7.1	0.32
RCA from non-coronary cusp	2	2.4	0.11
Anomalous LAD from RCA	2	2.4	0.11
Total — any anomaly of origin	85	100	4.59
Total — excluding high/anterior RCA and separate LAD–LCx ostia	24	28.2	1.30

*RCA* right coronary artery, *LAD* left anterior descending artery, *LCx* left circumflex artery, *RCC* right coronary cusp, *LCC* left coronary cusp

### Coexistent atherosclerotic coronary artery disease

Significant coronary artery disease (CAD; ≥ 50% stenosis) was present in 70 of 85 anomaly patients (82.4%) — not significantly different from the non-anomaly group (76.9%; *p* = 0.30). Single-, double- and triple-vessel disease occurred in 29 (34.1%), 23 (27.1%) and 14 (16.5%) anomaly patients respectively, and left main plus multivessel disease in two (2.4%) patients; 15 (17.6%) had normal/nonobstructive coronaries.

### Procedural variables and catheter burden

Procedural variables are compared in Table 3. Radial access was the default in both groups (62.4% vs. 72.9%), but cross-over to femoral access was more than three-fold higher in the anomaly group (16.5% vs. 5.0%; *p* < 0.001). Anomaly patients required substantially more diagnostic catheters per case (mean 2.20 ± 0.97 vs. 1.23 ± 0.44; *p* < 0.001), with ≥ 2 shapes used in 78.8% versus 22.7% (*p* < 0.001) and ≥ 3 shapes in 29.4% versus 0.6% (*p* < 0.001) — a roughly 50-fold gradient. Among patients undergoing PCI, a change of guide catheter was required in 23 of 52 anomaly patients (44.2%) versus 66 of 1,022 non-anomaly PCIs (6.5%; *p* < 0.001).

Exploratory multivariable logistic regression for access crossover, with coronary anomaly, age, sex, clinical presentation and PCI performance as covariates, confirmed that the presence of a coronary anomaly was a strong and statistically independent predictor (adjusted OR 3.92, 95% CI 2.05–7.52, *p* < 0.001), alongside PCI performance (adjusted OR 14.09, 95% CI 6.50–30.56, *p* < 0.001) and older age (per-year adjusted OR 1.03, 95% CI 1.01–1.05, *p* = 0.010). For guide-catheter change and complications, the number of events was insufficient to support multivariable modelling.

**Table 3:** Procedural variables and complications

Procedural variable	Anomaly (*n* = 85)	No anomaly (*n* = 1,765)	*P*
Radial-only access, n (%)	53 (62.4)	1,290 (73.1)	–
Femoral-only access, n (%)	18 (21.2)	386 (21.9)	–
Cross-over from radial to femoral, n (%)	14 (16.5)	89 (5.0)	< 0.001
Mean diagnostic catheters per case	2.20 ± 0.97	1.23 ± 0.44	< 0.001
≥ 2 diagnostic catheters used, n (%)	67 (78.8)	400 (22.7)	< 0.001
≥ 3 diagnostic catheters used, n (%)	25 (29.4)	11 (0.6)	< 0.001
Obstructive CAD on angiography, n (%)	70 (82.4)	1,357 (76.9)	0.30
Any major procedural complication, n (%)	4 (4.7)	47 (2.7)	0.43
PCI performed, n (%)	52 (61.2)	1,022 (57.9)	0.53
Change of guide catheter (of PCI cases), n (%)	23 / 52 (44.2)	66 / 1,022 (6.5)	< 0.001

*PCI* percutaneous coronary intervention, *SD* standard deviation

### Engagement route and the catheter achieving definitive engagement

The procedure was completed by the radial route only, in the majority of anomaly patients who were attempted by radial access (53 of 67; 79.1%), confirming that most anomalies of origin can be engaged radially despite their aberrant ostial location. While as 14 (14 of 67 attempted radial; 20.9%) required crossover from the radial to femoral route, 18 underwent the procedure via femoral route directly. The diagnostic catheter achieving definitive selective engagement, by anomaly subtype, is shown in Table 4.

**Table 4:** Diagnostic catheter achieving definitive^*^ selective engagement, by anomaly subtype

Anomaly subtype	*N*	Catheter achieving definitive engagement, *n* (%)
Anomalous high/anterior RCA from RCC	47	JR 26 (55.3); Tiger 10 (21.3); AR 6 (12.8); AL 3 (6.4); MP 1 (2.1); 3DRC 1 (2.1)
Separate ostia of LAD and LCx^**^	14	JL 7 (50.0); Tiger 6 (42.9); AL 1 (7.1)
Anomalous LCx from RCC or RCA^***^	14	JR 9 (64.3); AR 2 (14.3); AL 1 (7.1); Tiger 1 (7.1); MP 1 (7.1)
RCA from LCC	6	JL 3 (50); AL 2 (33.3); IMA 1 (16.7)
RCA from non-coronary cusp	2	AR 1 (50.0); JR 1 (50.0)
Anomalous LAD from RCA	2	JR 2 (100)

^*^Final catheter that selectively engaged the anomalous ostium, after any earlier failed attempts

^**^Catheter that engaged the LAD ostium, LCx opacified by non-selective sinus injection or catheter manipulation

^***^Catheter that opacified the anomalous LCx — either by selective engagement in the RCC, or via engagement of the RCA when the LCx arose as a proximal RCA branch

*RCA* right coronary artery, *LAD* left anterior descending artery, *LCx* left circumflex artery, *RCC* right coronary cusp, *LCC* left coronary cusp, *JR* Judkins Right, *JL* Judkins Left, *AR* Amplatz Right, *AL* Amplatz Left, *MP* multipurpose, *IMA* internal mammary artery, *3DRC* three-dimensional right curve

### Diagnostic catheter burden by access strategy

To examine whether the catheter-burden difference was uniform across access strategies, we stratified the diagnostic catheter count by the three access patterns (Table 5). Among radial-only cases, the mean was 1.94 ± 0.85 in the anomaly group versus 1.01 ± 0.11 in the non-anomaly group (*p* < 0.001) — a near two-fold difference, as the default Tiger sufficed in virtually all normal-anatomy radial cases whereas anomaly cases almost invariably required an additional shape. Among femoral-only cases the means were 2.17 ± 0.38 versus 2.08 ± 0.17 (*p* = 0.08). The greatest burden occurred in cross-over cases − 3.14 ± 1.35 versus 1.17 ± 0.53 (*p* < 0.001), reflecting both the diagnostic challenge and the additional catheters used after switching access.

**Table 5:** Mean diagnostic catheter burden by access strategy

Access strategy	Anomaly (mean ± SD)	No anomaly (mean ± SD)	*P*
Radial only	1.94 ± 0.85	1.01 ± 0.11	< 0.001
Femoral only	2.17 ± 0.38	2.08 ± 0.17	0.08
Cross-over (radial → femoral)	3.14 ± 1.35	1.17 ± 0.53	< 0.001

*SD* standard deviation

### Guide catheter used for PCI by anomaly subtype

Of the 85 anomaly patients, 52 underwent PCI. The catheter achieving successful PCI is shown in Table 6. For a high or anterior origin of the RCA from the right coronary cusp, JR (42.8%), the Amplatz Right (AR, 32.1%) and Amplatz Left (AL, 25.0%) guides were the most frequently successful. Separate ostia of the LAD and LCx were almost always treated with an EBU guide (85.7%) with half size less for the LAD. An anomalous LCx from the RCC or RCA was treated with AL, AR or JR guides in equal proportion (33.3% each). For RCA from the left coronary cusp, the AL was the most successful guide (66.7%). The two patients with an RCA from the non-coronary cusp underwent PCI with a EBU or JR guide.

**Table 6:** Catheter used for PCI (successful guide catheter) by anomaly subtype

Anomaly subtype	PCI performed, *n* / total	Catheter achieving successful PCI, *n* (% of PCI cases)
Anomalous origin of RCA from RCC	28 / 47 (59.6%)	JR 12 (42.8); AR 9 (32.1); AL 7 (25.0)
Separate ostia of LAD and LCx	7 / 14 (50.0%)	EBU 6 (85.7); JL 1 (14.3)
Anomalous LCx from RCC or RCA	9 / 14 (64.3%)	AL 3 (33.3); AR 3 (33.3); JR 3 (33.3)
RCA from left coronary cusp	6 / 6 (100%)	AL 4 (66.7); EBU 1 (16.7); AR 1 (16.7)
RCA from non-coronary cusp	2 / 2 (100%)	EBU 1 (50.0); JR 1 (50.0)
LAD-from-RCA	0/2 (0%)	

*PCI* percutaneous coronary intervention, *RCA* right coronary artery, *LAD* left anterior descending artery, *LCx* left circumflex artery, *RCC* right coronary cusp, *JR* Judkins Right, *JL* Judkins Left, *AR* Amplatz Right, *AL* Amplatz Left, *EBU* extra back-up

### Procedural complications

Major procedural complications occurred in 4 of 85 anomaly patients (4.7%) versus 47 of 1,765 non-anomaly patients (2.7%; *p* = 0.43); no statistically significant difference was observed. The four events in the anomaly group were one peri-procedural death from refractory cardiogenic shock, two access-site haematomas (meeting BARC type ≥ 3), and one catheter-induced ostial dissection of an anomalous RCA sustained during AL guide catheter engagement for PCI, which was treated by ostial stenting.

## Discussion

This prospective single-centre study of 1,850 consecutive patients undergoing invasive coronary angiography extends the existing literature by providing subtype-resolved data on the catheters that engage anomalies of coronary artery origin, together with the access profile encountered in contemporary radial-predominant practice. The prevalence of anomalies of origin was 4.59% under an inclusive definition, falling to 1.30% when the two commonest benign anatomical variants (high or anterior RCA origin from the right coronary cusp, and separate ostia of the LAD and LCx) were excluded as these variants are not consistently classified as anomalies in earlier invasive angiography studies. The strict-definition figure is presented specifically for comparison and is in close agreement with the classical international invasive-angiography literature [3, 5, 12, 22, 24]. CT-based series typically report substantially higher figures because they include intrinsic anomalies — most importantly myocardial bridging — that are systematically under-diagnosed at invasive angiography [14–16]. Our deliberately restrictive definition, anchored in the Angelini classification of anomalies of origin [3, 25], isolates the subgroup of anomalies with direct procedural relevance and supports valid comparison with the invasive literature.

### Spectrum of anomalies of origin

An ectopic (high or anterior) origin of the RCA from the RCC dominated the spectrum, accounting for 55.3% of all anomalies. This is consistent with published literature, in which RCA-related anomalies outnumber left coronary anomalies by approximately three to one [3, 12, 22, 24]. A high anterior RCA take-off is clinically benign but the ostium is displaced away from the standard sinus position; the default radial Tiger frequently fails to engage the anomalous ostium, and the default femoral Judkins Right may not engage at the usual site requiring manipulations and forceful non-selective injections risking catheter-induced spasm or dissection [4, 25]. Separate ostia of the LAD and LCx (16.5%) are a benign variant [3, 27] requiring independent engagement of each vessel. Anomalous origin of the LCx from the RCC or proximal RCA (16.5%) is haemodynamically benign [26, 27] but is not opacified by a standard left coronary injection and required significant efforts in diagnostic angiography and PCI. The RCA arising from the LCC was identified in six patients (7.1%). When such a vessel courses between the aorta and the pulmonary trunk it is associated with myocardial ischaemia and sudden cardiac death; identification mandates CT or magnetic resonance (MR) characterisation of the course [6, 28, 26, 29, 30]. The remaining two subtypes were rare with RCA arising from the non-coronary cusp (*n* = 2; 2.4%) and an anomalous LAD arising from the RCA (*n* = 2; 2.4%). Single coronary variants in which one epicardial vessel arises from another, have been reported to pose distinct engagement and revascularisation challenges [31].

### Catheter burden, access strategy and procedural implications

The most clinically actionable finding is the striking gradient in catheter burden: anomaly patients required nearly twice as many diagnostic catheters as non-anomaly patients (2.20 vs. 1.23), with three or more diagnostic catheters used in 29.4% versus 0.6% in addition to frequent guide catheter change in the anomaly group (44.2% vs. 6.5%). This translates into increased ostial manipulation and in turn complications [4, 5, 32]. The diagnostic catheter burden is not uniform across access strategies. In radial-only cases, normal-anatomy patients were managed with a single Tiger in essentially every case (1.01), whereas anomaly patients required nearly two shapes (1.94). In femoral-only cases the comparison was attenuated by the floor effect of standard JL + JR practice (2.08 vs. 2.17, *p* = 0.08). The largest absolute burden occurred in cross-over cases (3.14 vs. 1.17), reflecting both diagnostic challenge and additional catheters used after switching access for PCI. Despite this, 53 of the 67 anomaly cases attempted radially (79.1%) were completed without crossover, confirming that a radial-predominant strategy remains appropriate when a dedicated catheter set is available [20, 33]. These findings sit alongside contemporary transradial data supporting the safety and patient-acceptability of the radial approach in a range of clinical settings [34, 35]. The subtype-directed catheter-selection reference presented in Table 7 is intended to translate our findings into a reference format for the operator. Recent comparative angiographic studies have similarly reported increased procedural complexity and catheter utilisation in patients with anomalies of coronary artery origin [36, 37].

**Table 7:** Diagnostic tips-and-tricks and catheter selection by anomaly subtype

Subtype	Diagnostic clue on standard injection	Best projection	First-line diagnostic catheter*	Rescue diagnostic catheter*	PCI guide catheter*
High/anterior RCA from RCC	Standard catheter fails to hook; catheter tip sits at unusual site	LAO 30–40° for RCA ostial plane, RAO 30–40° for the anterior location	Aortic root injection (pigtail); then JR 4 with clockwise torque	AR1 or AR2; AL 0.75 or MP-1 if AR fails	JR 4: AR or AL1 for radial coaxial support
Separate LAD and LCx ostia (absent LM)	Left system opacifies only one vessel on standard JL injection	RAO 30°/caudal 25° for LCx; AP cranial for LAD	JL 3 for LAD	JL 4.0 for LCx (separate cannulation)	EBU/CLS or JL 3.5 for LAD, JL 4.0 for LCx
Anomalous LCx from RCC or proximal RCA	LAD-only on left main injection; bald LCx territory	RAO 30°/caudal 25°	JR4; with clockwise rotation and slow withdrawal from RCA ostium if separate ostium	AR1 or AR2, AL1, or MP	JR, AL, AR guide with distal RCA wire for stability
RCA from LCC	JR/RCC injection fails at usual site; contrast reflux from left cusp; catheter tip near LM	LAO 30°; RAO cranial	JL 3	AL1, AR1 or AR2, JR4 with anterior rotation,	AL for coaxial radial support, EBU or JL
RCA from NCC	JR fails at usual RCC site; posterior aortogram opacifies NCC	RAO 30–40°	JR4 directed posteriorly in NCC	AR1 or MP	JR or AR-family
Anomalous LAD from RCA	Only LCx opacified on left main injection; bald LAD territory	AP or LAO cranial for LAD	JR3.5 or JR4 in RCC — opacifies both RCA and LAD in one injection	AL1 if JR fails to engage	Same catheter used for RCA engagement

^*^Catheter sizes shown are for right radial access. For femoral access, upsize left-coronary catheters (JL, EBU) by 0.5, downsize JR by 0.5–1.0, and leave Amplatz (AL, AR) and multipurpose (MP) shapes unchanged (size to aortic root)

*JR* Judkins Right, *JL* Judkins Left, *AR* Amplatz Right, *AL* Amplatz Left, *MP* multipurpose, *LAO* left anterior oblique, *RAO* right anterior oblique, *AP* anteroposterior, *RCC* right coronary cusp, *LCC* left coronary cusp, *NCC* non-coronary cusp, *LM* left main, *LAD* left anterior descending artery, *LCx* left circumflex artery, *RCA* right coronary artery, *PCI* percutaneous coronary intervention

### Distinction between anatomical variants and clinically significant anomalies

Not all findings included under “anomalies of coronary artery origin” carry the same clinical weight. Benign anatomical variants such as an ectopic (high or anterior) RCA origin from the RCC, separate ostial origins of the LAD and LCx, and a LCx from RCC/RCA are procedurally relevant because they defeat standard catheter shapes, but they are not usually associated with prognostically important interarterial or intramural courses. In contrast, anomalous aortic origin of a coronary artery from the opposite sinus with an interarterial course may carry ischaemic and sudden-death risk [26, 38, 39]. Multimodal imaging — particularly CT coronary angiography — complements invasive angiography by characterising vessel course when an interarterial variant is suspected [40, 41, 42]. Recognition of the anomaly at the index study also has implications for subsequent surgical planning, including graft strategy and coronary reimplantation techniques. This distinction matters for our findings, which relate to the mechanical task of engagement and not to the prognostic significance of specific vessel courses.

### Safety profile

Procedural complications were numerically higher in anomaly patients (4.7%) than in the non-anomaly group (2.7%) but did not reach statistical significance (*p* = 0.43). Given the small number of events, no confident statement can be made about whether anomaly patients carry an incremental procedural risk; our findings are best summarised as showing no significant difference in this cohort while acknowledging that the study was not powered for a rare-event safety comparison. This numerical excess, combined with the observation that anomaly patients require more catheters and more access crossovers, supports a cautious approach at the ostium — with early escalation to an anatomically appropriate catheter shape rather than repeated manipulation with a standard curve — as a reasonable operational principle.

### Limitations

First, the study was scoped to the mechanical task of engaging anomalous coronary ostia at index angiography; fluoroscopy time, contrast volume, radiation dose and total procedure duration were not part of the pre-specified data collection, and the increased catheter burden observed can only be inferred to translate into greater radiation and contrast exposure. Second, CT coronary angiography was performed selectively rather than in every anomaly patient, so the data do not permit a reliable cohort-wide distinction between benign and potentially malignant interarterial courses, and prognostic inferences should be interpreted with caution. Third, long-term clinical outcomes — mortality, repeat revascularisation and major adverse cardiovascular events — were not captured. Fourth, this is a single-centre study conducted under a defined institutional catheter protocol (default 5-Tiger for radial access and Judkins pair for femoral access), and the specific catheter-utilisation pattern may not be directly generalisable to centres with different default catheters.

### Future perspectives

Future work should focus on prospective multicentre registries capturing fluoroscopy time, contrast volume, radiation dose and systematic CT correlation, permitting direct quantification of procedural complexity. Larger datasets would support the development of a validated decision-support algorithm and inform the incorporation of anomaly recognition into interventional training curricula.

## Conclusions

Anomalies of coronary artery origin were identified in 4.59% of patients undergoing invasive coronary angiography (1.30% under a restricted definition) and were associated with a higher diagnostic catheter burden, more frequent access crossover, and more frequent guide-catheter change during PCI. An anomaly remained an independent predictor of access crossover after multivariable adjustment, though most radial-attempted cases were completed without crossover. Awareness of the diagnostic clue and subtype-directed catheter selection may aid efficient engagement.

## Data Availability

The data is available from the corresponding author on reasonable request.

## Abbreviations

3DRC: Three-dimensional right curve
AL: Amplatz Left
AR: Amplatz Right
CAD: Coronary artery disease
CLS: Contralateral left support
CT: Computed tomography
EBU: Extra back up
IMA: Internal mammary artery
JL: Judkins Left
JR: Judkins Right
LAD: Left anterior descending
LCx: Left circumflex
MP: Multipurpose
MR: Magnetic resonance
NSTEMI: Non-ST-elevation myocardial infarction
PCI: Percutaneous coronary intervention
RCA: Right coronary artery
SD: Standard deviation
STEMI: ST-elevation myocardial infarction
